# Relationship between Skin Temperature, Electrical Manifestations of Muscle Fatigue, and Exercise-Induced Delayed Onset Muscle Soreness for Dynamic Contractions: A Preliminary Study

**DOI:** 10.3390/ijerph17186817

**Published:** 2020-09-18

**Authors:** Jose I. Priego-Quesada, Carlos De la Fuente, Marcos R. Kunzler, Pedro Perez-Soriano, David Hervás-Marín, Felipe P. Carpes

**Affiliations:** 1 Research Group in Sport Biomechanics, Department of Physical Education and Sports, University of Valencia, 46010 Valencia, Spain; pedro.perez-soriano@uv.es; 2 Biophysics and Medical Physics Group, Department of Physiology, University of Valencia, 46010 Valencia, Spain; 3 Applied Neuromechanics Research Group, Laboratory of Neuromechanics, Federal University of Pampa, Uruguaiana, RS 97500-970, Brazil; delafuentte@gmail.com (C.D.l.F.); mrkjoia@yahoo.com.br (M.R.K.); carpes@unipampa.edu.br (F.P.C.); 4 Carrera de Kinesiología, Departamento de Ciencias de la Salud, Facultad de Medicina, Pontificia Universidad Católica de Chile, Santiago 7820244, Chile; 5 Centro de Salud Deportivo, Clínica Santa Maria, Santiago 7520380, Chile; 6 Unidad de Bioestadística, Instituto de Investigación Sanitaria La Fe, 46026 Valencia, Spain; bioestadistica@iislafe.es

**Keywords:** exercise recovery, infrared thermography, biceps brachialis, electromyography, physical exercise

## Abstract

Delayed onset muscle soreness (DOMS) indicates the presence of muscle damage and impairs force production and control. Monitorization of DOMS is useful to improving recovery intervention plans. The magnitude of DOMS may relate to muscle fatigue, which can be monitored by surface electromyography (EMG). Additionally, growing interest has been expressed in determining whether the skin temperature over a muscle group during exercise to fatigue could be a non-invasive marker for DOMS. Here we determine whether skin temperature and manifestations of muscle fatigue during exercise are correlated and can predict DOMS after concentric–eccentric bicep curl exercises. We tested 10 young adults who performed concentric–eccentric bicep curl exercises to induce muscle damage in the biceps brachialis to investigate the relationship between skin temperature and fatigue during exercise and DOMS after exercise. Muscle activation and skin temperature were recorded during exercise. DOMS was evaluated 24 h after exercise. Data analysis was performed using Bayesian regression models with regularizing priors. We found significant muscle fatigue and an increase in skin temperature during exercise. DOMS was observed 24 h after exercise. The regression models showed no correlation of changes in skin temperature and muscle fatigue during exercise with DOMS 24 h after exercise. In conclusion, our preliminary results do not support a relationship between skin temperature measured during exercise and either muscle fatigue during exercise or the ability to predict DOMS 24 h after exercise.

## 1. Introduction

Delayed onset muscle soreness (DOMS) can be experienced from 24 h to 7 days after intense exercises involving eccentric muscle actions, as well as in response to sudden increases in workload and muscle fatigue [1]. The intensity and duration of the exercise [1,2], type of contraction (concentric or eccentric) [3], and type of exercise [4] also play a role in DOMS. DOMS impairs functionality and neuromuscular performance [5]. For this reason, predicting it based on the characteristics of the exercise session could help to improve recovery interventions such as massage, stretching, non-steroidal anti-inflammatory drugs, cryotherapy, and compression, among others [6].

Fatigue plays a main role in muscle damage. Although muscle fatigue represents a complex phenomenon [7], in the context of the present work it may be defined as the decrease in force or power capacity resulting in task failure due to incapacity for contractile activity [8,9,10], which can be described by the mean or peak of spectrum compression of neuromuscular electrical activation recorded under muscle fatigue [7,10,11]. Eccentric actions in general result in a lower magnitude of muscle activation, while fatigue due to submaximal exercise will increase muscle activation and decrease the firing rate [12]. A possible interaction between DOMS and neuromuscular activation could exist because the main conditions leading to DOMS, which include eccentric actions and muscle fatigue, are detectable through neuromuscular activity patterns.

While the neuromuscular activity during exercise is often used to estimate adaptations, in recent years, growing interest has arisen in the use of infrared thermography to record information during exercise and to evaluate acute responses [13,14]. Skin temperature has become popular in sports and clinical contexts with the aim of predicting the risk of injury [15,16]. The relationship between neuromuscular activation and skin temperature remains unclear. Bartuzi et al. [17] observed skin temperature increases for biceps brachialis during isometric muscle actions performed until fatigue (quantified by myoelectrical manifestations of the root-mean-square, mean power frequency, and median frequency). Priego Quesada et al. [18] found that participants with more efficient neuromuscular activation during cycling (expressed by greater overall neuromuscular activation and a reduced low-frequency component for vastus lateralis activation) showed a better adaptive thermoregulatory response through a lower increase in skin temperature after cycling exercise. This result may also relate to the local monitoring of heat produced by muscular contractions. Considering that muscle contraction produces heat and that sustained exercise leads to an increase in body temperature [19], heat transfer to the environment could influence skin temperature. In this sense, resistance exercises may cause lower sweat production, resulting in skin temperature increases, which could result in a direct association between muscular contractions and skin temperature response, contrary to what happens during other exercises (e.g., cycling and running) that result in whole-body sweating [13]. Therefore, investigating the relationship between neuromuscular activation and skin temperature during exercise could help us to understand the evolution of skin temperature during different physical exercises and intensities [13]. The measurement of skin temperature by infrared thermography can also provide non-invasive information about the efficiency of the thermoregulatory system in dissipating heat [18]. In a previous study, electrical manifestations of muscle fatigue (i.e., the mean and peak frequency of electromyography signals) were not considered in relation to skin temperature [20].

Associations between electrical manifestations of muscle fatigue, skin temperature, and DOMS have not been investigated in previous studies. The possible associations between electrical manifestations of muscle fatigue and DOMS and between electrical manifestations of muscle fatigue and skin temperature could lead to an association among the three outcomes. For example, it has been suggested that higher DOMS is related with higher physical fitness level [21,22], in the same way that differences in skin temperature responses have been observed to depend on physical fitness [13,18,23,24]. Although higher physical fitness could be associated with lower skin temperature increase due to a better heat dissipation response [18], it could be also associated with quicker [24] and higher increase of skin temperature for a higher capacity of heat production [23]. These possible correlations determined during exercise would help to understand the relevance of practical applications of infrared thermography for monitoring exercise-induced adaptations in different contexts (i.e., sport medicine, rehabilitation, injury prevention, etc.).

In this preliminary study, we set out to verify whether skin temperature and frequency manifestations of fatigue during small muscle mass exercise are related and can predict DOMS. We hypothesized that skin temperature and frequency manifestations of muscle fatigue would correlate due to the higher heat production in response to exercise. We also hypothesized that if it is possible to predict DOMS, the intensity of muscle fatigue manifestation (e.g., decrease in peak frequency) could be the main predictor variable, with higher DOMS being associated with increased muscle fatigue manifestation.

## 2. Materials and Methods 

### 2.1. Experimental Design

**Figure 1:**
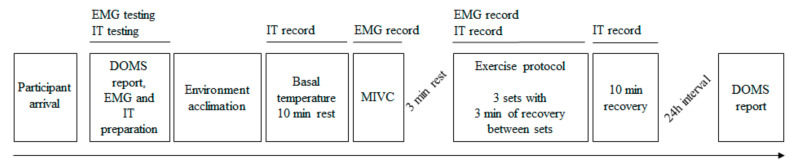
Flowchart of the study protocol. EMG: surface electromyography; IT: infrared thermography; MIVC: maximal isometric voluntary contraction; DOMS: delayed onset muscle soreness.

We undertook a cross-sectional study including active men who performed a series of biceps curl exercises at submaximal load until exhaustion. During the entire exercise session, skin temperature and surface electromyography data were recorded. DOMS was evaluated before and 24 h after the exercise. Figure 1 depicts the experimental design.

### 2.2. Participants

For this preliminary study and based on previous results [18], to detect a bivariate correlation equal to 0.76, a minimum sample size of 9 participants was estimated using a power of 80% and α error of 5% (G*Power 3 software, University of Düsseldorf, Düsseldorf, Germany). Therefore, ten volunteer participants were recruited to take part in this study. They signed a written consent form. The ethics committee from the Universidade Federal do Pampa approved this study (Code: 60376216.4.0000.5323; Date of approval: 23 August 2017). They were all men of the following (mean ± standard deviation): age 27 ± 4 years old, height 179 ± 6 cm, body mass 75 ± 9 kg, bicep skinfold 3.7 ± 1.4 mm, non-smokers, and physically fit through performing whole-body exercises such as running, cycling, and court sports on average 5 ± 2 days per week. In the previous 12 months, none had experienced injury or extreme muscle soreness. Half of the participants were familiar with upper limb strength training. All participants identified themselves as preferring the right upper limb in accordance with Waterloo Handedness Questionnaire [25].

### 2.3. Procedures

Participants were requested to avoid intense exercise in the 24 h prior to the exercise protocol, as well as to avoid intake of alcoholic or caffeine-enriched beverages, sunbathing, physiotherapy treatments (e.g., massages or cryotherapy), and the use of skin cream—all recommendations to minimize any influence on skin temperature measurements [14,26]. Another instruction was to avoid large meals in the 4 h period prior to the test [14]. Data were collected from one participant at a time. After arriving at the laboratory, demographic data were collected, bicep skinfolds were measured using a caliper (Holtain LTD., Crymych, UK) following the protocol of the International Society for the Advancement of Kinanthropometry (ISAK) [27], and the details of the protocol were explained to the participant. They completed the exercise protocol between 09:00 and 12:00 in the morning in order to avoid circadian effects [28,29]. The exercise protocol started with a standardized warm-up including stretching of the shoulder, elbow, and wrist muscles and active flexion and extension of the shoulder, followed by bicep exhaustion at submaximal load [30]. The warm-up lasted ~1 min. The participant was then requested to perform 3 sets of elbow flexion–extension (bicep curl exercise) until exhaustion, with the preferred arm. The load was set at 7% of individual body mass and adjusted using a bar bell and known weights. This load was adapted from a previous study using an absolute value that we estimated as corresponding to 7% of the participants’ body mass [30]. The movement should be performed at the full elbow range of motion, while seated, with the trunk aligned to the vertical and at the frequency of 20 beats per minute following auditory feedback from a metronome [31]. Each audio beat was associated with the end of the elbow flexion. The sets were performed until the participant was no longer able to perform the full range of motion. A 3 min recovery period was conducted between each of the 3 sets. 

During the exercise series, neuromuscular electrical activation and skin temperature were continuously recorded. To record surface electromyography (EMG) signals from biceps brachialis, the participants’ skin was prepared according to Surface ElectroMyoGraphy for the Non-Invasive Assessment of Muscles (SENIAM) recommendations [32]. EMG measurements referred to the preferred arm and were sampled at 1000 Hz, Common Mode Rejection Ratio (CMRR) of 110 dB, and bandwidth between 15 and 500 Hz using a wireless electromyography device (Mega Electronics, Kuopio, Finland). The EMG signal was pre-amplified to a factor of 1000 and converted from analogue to digital using a 14-bit A/D converter (National Instruments, New South Wales, Australia). The Ag/AgCl electrodes employed were disposable surface passive electrodes placed on the skin over the belly of biceps brachialis [32]. Two electrodes were placed with an inter-electrode distance of 20 mm and parallel to the muscle fiber orientation. A third electrode served as the electrical reference and was placed at the acromion protuberance. A small piece of athletic tape was used to prevent the electrode and wireless transmitter from moving [32]. A maximal voluntary isometric contraction for elbow flexion at approximately 90° was performed against manual resistance from one researcher before the exercise protocol started in order to provide the data for EMG normalization [33,34,35]. 

After EMG preparation and before starting the exercise, there was a 10 min rest period to allow the necessary acclimatization for collecting infrared thermography data [36]. When the 10 min period was over, a baseline image of the participant was recorded. Further data were collected during the entire exercise protocol using infrared thermography from the region of interest in the preferred and the non-preferred arm. The non-preferred arm was considered as a control. The images were continuously recorded using a commercial software package (FLIR Tools+, FLIR, Wilsonville, OR, USA) with an emissivity factor of 0.98 [37] under controlled environmental temperature and air humidity (21.3 ± 1.4 °C and 28.3 ± 7.0%) using two air conditioning units (model PSA-RP140GA, Mitsubishi Electric, Tokyo, Japan). In addition, the reflected temperature (21.2 ± 0.4 °C) was measured using the standard method ISO 18434-1:2008. The infrared images were recorded throughout the exercise at 7.5 Hz using an infrared camera with a resolution of 320 × 240 pixels, noise equivalent temperature difference (NETD) of <0.05 °C, and measurement uncertainty of 2 °C (FLIR E-60, Flir Systems Inc., Wilsonville, OR, USA). The camera was turned on 10 min before the recordings and positioned at 1.5 m high and 1.5 m far from the participant with the lens parallel to the region of interest and perpendicular to the plane of interest. An antireflective panel was positioned behind the participant to minimize the effects of infrared radiation reflecting from the wall [26]. Before starting the study, the camera was calibrated using a black body (BX-500 IR Infrared Calibrator, CEM, Shenzhen, China). The Thermographic Imaging in Sports and Exercise Medicine checklist (TISEM) was used to ensure that all the recommended procedures were followed [14]. 

Before and 24 h after the exercise, DOMS was quantified by the same experimenter using a Borg CR-10 visual scale [38] in which 0 (zero) meant no pain and 10 (ten) represented very strong pain. The participant was questioned about the pain sensation during passive flexion and extension of the elbow joint [39].

### 2.4. Data Processing

To obtain the frequency manifestation of fatigue (median and peak frequencies) during each series, at different moments of the exercise (when the elbow was at full extension considering 25%, 50%, 75%, and 100% of the total repetitions in each series), the EMG signals were analyzed offline using custom codes written in Matlab (2016a, Mathworks Inc., Natick, MA, USA). The signals in the time domain were mean-centered by subtracting the mean value and filtering using a Butterworth zero-lag second-order finite impulse response filter with a band pass of 20 to 500 Hz [32]. The Threshold-based method and Teager–Kaiser energy operator, full-wave rectified and zero-lag lowpass filtered, were used for burst detection [40]. The bursts were then concatenated for analysis. The first and last bursts were not considered for analyzing the burst most synchronized with the metronome. The EMG signals in the time domain were treated using a fast independent component algorithm that is recommended by Hyvärinen & Oja [41] to avoid the inclusion of non-biological components in the signal based on a linear statistical decomposition of an independent component. The EMG signals were then reconstructed. The frequency spectrum was determined using the fast Fourier algorithm [42] extracting the median and peak of frequencies of a non-overlapped rectangular window of 500 ms from the Fourier spectrum. Finally, the slopes of the median and peak frequencies of each and the entire series were obtained using a linear regression method. The coefficients of fatigue were normalized between 0 and 1 with respect to the first value. The median frequency (MDF) was determined by the slope of linear regression for each series. This is a continuous variable, and the 50th percentile of the Fourier spectrum was used. The slope of linear regression for each series determined the peak frequency. This is a continuous variable, and we used the maximal value of the Fourier spectrum. The “spectrum compression” is an electrical manifestation of muscle fatigue where more negative slopes in the regression represent greater compression of the frequency spectrum, and higher frequency slopes have a high association with muscle fatigue [7,11]. 

**Figure 2:**
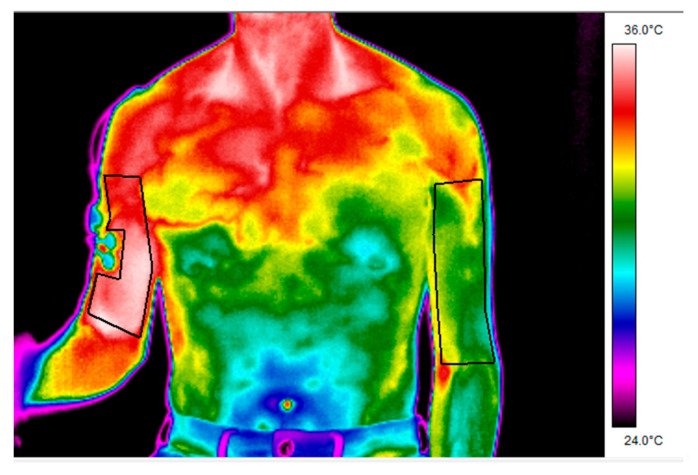
Regions of interest in which skin temperature was determined using infrared thermography.

The mean skin temperature was determined bilaterally for the exercised arm and the non-exercised control arm, considering the biceps brachialis region of interest (Figure 2) at the same time as the EMG data were being processed (25%, 50%, 75%, and 100% of the total repetitions in each series). Thermal images were recorded using a thermographic software package (ThermaCam Researcher Pro 2.10 software, FLIR, Wilsonville, OR, USA). The sizes of the regions of interest were 1640 (208) and 2345 (417) pixels for the exercised and control arms, respectively.

### 2.5. Statistical Analysis 

Data are summarized herein using the mean (standard deviation) for continuous variables and absolute and relative frequencies for categorical variables. The association of temperature with the number of bicep curl repetitions, series, and arm was modelled using a multivariable linear regression mixed model including a three-way interaction between number of repetitions, series, and arm. To assess the association between DOMS and upper limb strength training, number of repetitions, peak slope, and temperature slope (coefficient obtained by the linear regression mixed model), an ordinal regression model was used. In order to avoid overfitting, the model was penalized using a horseshoe prior in a Bayesian setting [43]. The relationships of MDF and peak frequency with temperature, number of repetitions, and series were assessed using a beta regression model including the three covariates and an interaction between repetition number and series. Odds ratios (ORs), 95% credibility intervals (95% CIs), and evidence ratios are provided. The assumptions of the linear model and beta regression were assessed by residual plots. In the case of the ordinal regression model, proportional odds were assessed using the Brant test. In all cases, residual plots showed no evidence of a large deviation from the assumptions. Finally, bivariate Pearson correlation tests were performed to examine the relationship between variations (difference between the end of the test and basal measure) in neuromuscular activation, skin temperature of the dominant limb, and DOMS. The significance level was set at α = 0.05. All statistical analyses were performed using R (version 3.5.3) and R packages brms (version 2.8.0) and glmmADMB (version 0.8.3.3).

## 3. Results

### 3.1. DOMS

The participants reported no DOMS before exercise (score of 0). At 24 h after exercise, the average DOMS score was 6.2 ± 3 points. In the adjusted Bayesian ordinal regression model for DOMS, higher values of bicep skinfold were associated with lower DOMS values (OR = 0.24, 95% CI [0.06, 0.97], evidence ratio = 56.1), and a higher number of repetitions was associated with a higher DOMS score (OR = 0.06, 95% CI [1, 1.15], evidence ratio = 19.0). No evidence of association with DOMS for either skin temperature response or frequency manifestations of muscle fatigue during exercise was found (OR = 0.83, 95% CI [0.025, 12.18], evidence ratio = 1.12 and OR = 1.04, 95% CI [0.037, 23.5], evidence ratio = 1.01, respectively). In the same way, considering bivariate Pearson correlations, DOMS was not found to correlate with variation in the MDF (*r* = 0.02, *p* = 0.95), peak frequency (*r* = −0.24, *p* = 0.51), or skin temperature of the dominant limb (*r* = 0.09, *p* = 0.82).

### 3.2. Neuromuscular Fatigue and Temperature Response to Exercise

Participants performed 43 ± 13, 27 ± 5, and 24 ± 9 repetitions in the first, second, and third series, respectively.

**Figure 3:**
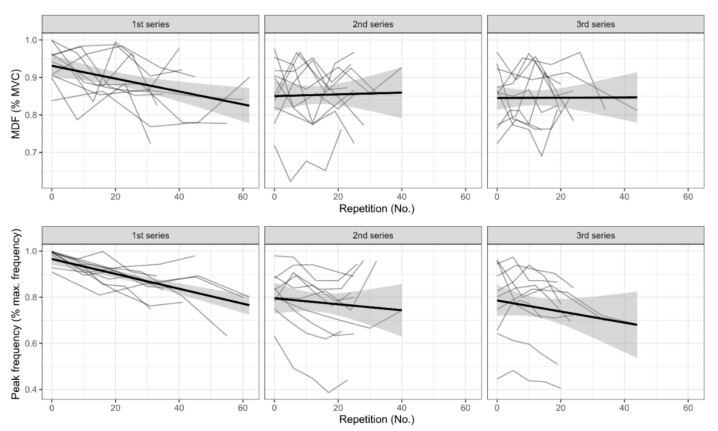
Neuromuscular activation responses during exercise during the three series. A linear trend line with a 95% credibility interval (CI) has been added to each series plot. MDF is the median frequency and MVC is the maximum voluntary contraction.

The beta regression model showed that MDF values were lower in the second (OR = 0.37, 95% CI [0.23, 0.58], *p* < 0.001) and third series (OR = 0.36, 95% CI [0.22, 0.59], *p* < 0.001) compared with the first one (Figure 3). The effect of number of repetitions differed between the series, with a negative trend in the first series and a neutral slope in the second and third series (main effect for repetition number: OR = 0.98, 95% CI [0.96, 0.99], *p* < 0.001; interaction between second series and repetition number: OR = 1.02, 95% CI [1.00, 1.04], *p* = 0.018; interaction between third series and repetition number: OR = 1.02, 95% CI [1.00, 1.04], *p* = 0.044). 

The peak frequency behaved similarly, showing lower values in the second (OR = 0.14, 95% CI [0.09, 0.23], *p* < 0.001) and third series (OR = 0.15, 95% CI [0.09, 0.25], *p* < 0.001) compared with the first series (Figure 3). As in the case of MDF, the effect of repetition number was different between the series (main effect for repetition number: OR = 0.96, 95% CI [0.95, 0.97], *p* < 0.001; interaction between second series and repetition number: OR = 1.03, 95% CI [1.01, 1.05], *p* = 0.001; interaction between third series and repetition number: OR = 1.02, 95% CI [1.00, 1.03], *p* = 0.054).

These results show the onset of the fatigue process in the participants. However, no evidence for an association between frequency manifestations of fatigue (MDF and peak frequency) and skin temperature response was found in our regression models or bivariate correlations. 

**Figure 4:**
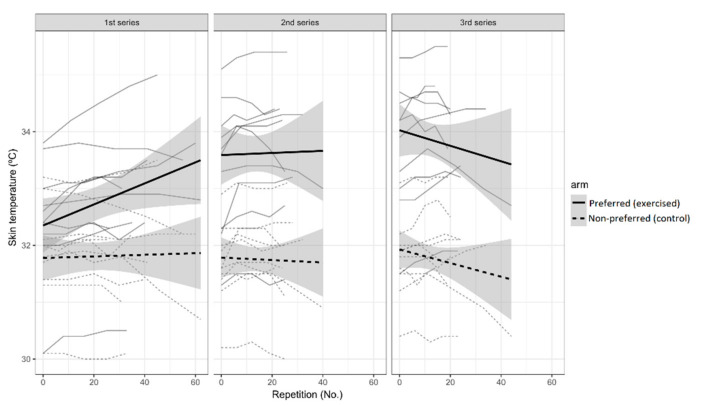
Skin temperature responses during exercise for the three series in both arms. For each arm, a linear trend line with a 95% CI has been added to each series plot.

The linear regression model showed that skin temperature significantly increased in the exercised region with the repetition number in the first series (slope = 0.013, 95% CI [0.01, 0.02], *p* = 0.004) (Figure 4). The non-exercised arm did not increase in skin temperature (interaction between arm and repetition number: slope = −0.017, 95% CI [−0.03, −0.01], *p* < 0.001). In the second series, the effect of repetition number in the exercised arm did not differ (interaction between repetition number and second series: slope = −0.009, 95% CI [−0.021, 0.004], *p* = 0.18). However, the 95% confidence interval for the interaction between repetition number and third series does not include 0 (slope = −0.018, 95% CI [−0.031, −0.006], *p* = 0.005), so it is possible to interpret this as indicating that there is a change towards negative values in the trend of skin temperature versus repetitions between the first and the third series, with a decrease of skin temperature in the exercised arm as the number of repetitions increased. The bicep skin temperature in the exercised arm presented a continuous increase of skin temperature in the first series, an almost flat behavior in the second series, and a decrease in the third one. On the other hand, the contralateral arm did not show any trend in the average temperatures (*p* > 0.05). 

## 4. Discussion

In this preliminary study, we determined whether skin temperature and frequency manifestations of muscle fatigue during small muscle mass exercise are correlated and can predict DOMS after exercise. Myoelectrical manifestations showed that fatigue was present at the end of exercise as indicated by lower MDF and peak frequency than at the beginning, but the behavior of the activation was not related to the changes in skin temperature in the exercised biceps. In addition, although muscle soreness was higher 24 h after exercise, DOMS was not predicted by the frequency myoelectrical manifestations of fatigue and skin temperature responses during the exercise. Therefore, both stated hypotheses were not confirmed. Our results have important implications in the field of clinical use of infrared thermography, where skin temperature data are used to assess exercise effects. We recommend caution when using skin temperature to monitor the exercise responses of small muscles.

During muscle contractions, must of the energy generated is heat (>70%) that is transferred to the skin by conduction between tissues or by convection through capillary blood flow [19,44]. Therefore, we considered that work production by the muscles would influence skin temperature, and as the exercise was performed to fatigue, the higher neuromuscular activation in the time course of fatigue would be related to changes in skin temperature due to heat production in the muscles. No evidence for this association was found in our experiment using small muscle mass exercise. One could argue that changes in skin temperature by the end of the exercise may depend on the influence of sweat. However, we consider that for an exercise involving a small muscle group (concentric–eccentric bicep curl exercise), this relationship would be less influenced by sweat responses than those in previous studies [18,45]. DOMS was not related with the frequency of myoelectrical manifestations of fatigue and skin temperature during the exercise. Although tissue damage associated with DOMS could result in an inflammatory response and an increase of skin temperature [16,46], this process is not instantaneous. At least 6 h was suggested as being necessary to possibly detect any inflammatory response to exercise [47]. We measured the temperature before, during, and 10 min after exercise. When skin temperature was measured at the same time as DOMS in a previous study, no relationship was found [20]. Our data cannot support the use of skin temperature to predict the magnitude of muscle damage resulting from exercise and causing DOMS. 

Few studies have assessed the relationship between muscle activation and skin temperature responses to exercise. Bartuzi et al. [17] observed an inverse relationship between biceps brachii activation and skin temperature during fatigue resulting from isometric contractions. Similarly, Kuniszyk-Jóźkowiak et al. [48] found an inverse relationship between skin temperature and median and mean frequencies of activation in the rectus femoris muscle in volleyball players required to sustain 70% of peak torque in the joint for as long as possible. Priego Quesada et al. [18] showed that during an incremental cycling exercise, participants with higher overall neuromuscular activation and lower frequency content in the activation for vastus lateralis (suggesting better efficiency in neuromuscular activation) presented lower increases in skin temperature (suggesting better thermoregulatory responses). It was further observed that cyclists with a lower percentage of maximum activation of the vastus lateralis while exercising at the intensity of their anaerobic threshold presented a lower increase in skin temperature [23].

The relationships between myoelectrical manifestations of fatigue and skin temperature previously reported [17,18,48] for exercises with higher workload were not observed here, in which dynamic muscle contractions were performed at a workload of 7% of body weight and movement frequency of 20 beats per minute. We assumed that sweat was not important in our study, unlike in previous studies [18,23]. It is important to mention that although this assumption was observed in a qualitative way, a previous study assessing squat exercises also mentioned that sweat was negligible during the experiment [49]. We recognize that this can be considered a limitation in our study because sweat production was not measured. Variability in heat production and gross efficiency among the participants can also influence skin temperature responses to exercise [50,51]. As mentioned above, the heat produced can reach the skin either by conduction or by convection [19,44]. Thermal conduction is a slow process that can vary between participants depending on the muscle–skin thermal gradient and thickness, and it can be hindered by insulating tissues such as fat tissue [19,52]. The capacity to modify skin blood flow (vasoconstriction or vasodilation) depending on the muscle blood needs or thermal stress could also differ between participants [24,53].

Two possible thermoregulatory explanations could explain the skin temperature response observed. Firstly, if there is a demand for energy from the muscles and non-thermal stress, peripheral vasoconstriction can occur in order to prioritize the blood supply to the exercised muscles [54]. This would result in lower heat transference from muscle to skin as observed in the second and third series, when skin temperature did not increase, but decreased in the final stage of muscle fatigue. Previous studies also found decreases in skin temperature during resistance exercise that they explained by peripheral vasoconstriction [49,53]. This peripheral vasoconstriction could also explain the reduction of the skin temperature in the contralateral arm. Secondly, a possible increase in body sweat production could also explain this skin temperature response in the third series. This increase in sweating due to thermal needs would also be accompanied by an increase in peripheral vasodilation [13]. In addition, Cheung and Sleivert [55] suggested a strong role of skin temperature in force production after knee isokinetic exercise with heating or cooling room temperature, where both physiological variables decreased, as may have occurred during the third series in our experiment. A non-linear behavior may exist for skin temperature in response to exercise, while frequency myoelectrical manifestations decreased from the beginning to the final stage of fatigue in a linear manner. 

Regression models showed that participants with lower bicep skinfolds and a higher total number of repetitions performed during the test were more likely to be subject to DOMS 24 h after exercise. These variables could be considered as participants’ parameters related with their physical condition (strength capacity) and performance (total number of repetitions in the bicep curl test until exhaustion). Previous studies suggested that participants with a higher force capacity have increased creatine kinase levels and, therefore, DOMS [21]. In exercises involving force generation, mainly the fast-twitch fibers are damaged, which could increase the DOMS of participants in better physical condition [56]. A different methodological approach was suggested as the reason for the discrepancies between studies in terms of the positive or negative relationship between physical fitness and DOMS [57]. In our study, each set was performed until failure, and a direct relationship could be due to the higher capacity of these participants to support pain in the last repetitions [58], resulting in greater tissue damage. Another factor that may have contributed to the difference in the number of repetitions is the technique used in the movement, where familiarity with the exercise could have an influence on participants’ performance.

As a preliminary study, there are different limitations. We considered a small sample of men. The behavior of these variables in women may vary due to specific characteristics of thermoregulation. Although we considered in our study that the presence of sweat was negligible, it may have affected the temperature responses of the skin during the exercise. Moreover, the measurement of muscle damage by blood markers, skin blood flow, sweat production, and core and muscle temperature would also assist in interpreting the results. We measured DOMS 24 h after exercise, and more severe symptoms could have been observed 48 h later. Finally, we analyzed the mean skin temperature of a ROI in the arm, and future studies could address alternative methods such as tmax analysis [59,60]. Future studies may also consider the quantification of DOMS by objective measures like the mechanical pain threshold.

## 5. Conclusions

Delayed onset muscle soreness 24 h after small muscle mass exercise did not correlate with skin temperature response and frequency myoelectrical manifestations of fatigue during dynamic contractions. Our preliminary results do not support the use of skin temperature measurements during exercise to predict DOMS 24 h after exercise.
